# EquityRx Tank: A Shark Tank-Inspired, Game-Based Workshop to Build Persuasive Communication and Collaboration Skills in Health Professions Education

**DOI:** 10.1007/s40670-026-02685-9

**Published:** 2026-02-27

**Authors:** Kristina Kaljo, Abbey Kruper

**Affiliations:** 1 Department of Obstetrics and Gynecology, University of Wisconsin, Madison, WI USA; 2 Present Address: Department of Obstetrics and Gynecology, Medical College of Wisconsin, Milwaukee, WI USA

**Keywords:** Game-based learning, Health professions education, Interprofessional collaboration, Professional competencies

## Abstract

Health professions education often prioritizes content mastery over essential competencies such as communication, advocacy, and interprofessional collaboration. EquityRx Tank, a Shark Tank-inspired, game-based workshop, was developed to foster persuasive communication and collaboration skills. Grounded in Understanding by Design and Experiential Learning Theory, EquityRx Tank was implemented as a standalone 90-minute session within a six-week summer enrichment program. The workshop was delivered across five cohorts (2019–2023; *n* = 71) and enrolled primarily undergraduate learners, many of whom self-identified as members of groups underrepresented in medicine and biomedical research. Participants represented varied racial/ethnic identities, geographic backgrounds, and first-generation college student status. Learners rated the workshop highly (mean 9.44/10). Reflexive thematic analysis of written feedback identified themes of satisfaction, engagement, productive discomfort, collaboration, and perceived skill development. EquityRx Tank offers a resource-light, reproducible framework with tangible instructional materials that can be adapted for enrichment programs, interprofessional curricula, and faculty development.

**Supplementary Information:**

The online version contains supplementary material available at 10.1007/s40670-026-02685-9.

## Introduction

Health professions education faces the persistent challenge of balancing content-heavy curricula with intentional development of essential professional competencies such as communication, advocacy, and interprofessional collaboration [1, 2]. Learners are expected to demonstrate critical thinking, adapt to dynamic environments, and communicate effectively with diverse audiences. Yet traditional didactic instruction remains the dominant approach, and these passive methods often fall short in cultivating competencies widely recognized as vital for success across all health professions [3–5].

The underemphasis of these competencies is not new. Reviews of professionalism training in medical education highlight a consistent gap in the teaching and assessment of interpersonal and persuasive communication [1, 6]. Learners themselves report variability in opportunities to practice authentic communication and decision-making, noting that these experiences are too often incidental rather than intentional [7]. Without structured opportunities, learners risk graduating with gaps in their ability to advocate, collaborate, and effectively lead in healthcare and community settings, and dedicated opportunities that build skills in communication and advocacy [8–11].

Active learning approaches offer one potential solution [12, 13]. When guided by clear objectives, active strategies such as problem-based, inquiry-driven, and game-based learning have been shown to enhance engagement and support learner motivation [14]. Game-based instructional strategies, in particular, provide opportunities for experimentation in a risk-free environment while fostering healthy competition and collaboration [14–17]. Recent syntheses suggest gamification can support engagement and learning in health professions education, with effects that vary depending on alignment between design elements and intended learning goals [18].

Despite these advantages, game-based instructional approaches remain underutilized, and when applied, they often reinforce content mastery rather than intentionally cultivate and enhance professional competencies. This gap serves as a space for innovative, theory-driven approaches that use game-based learning to address communication, advocacy, and interprofessional collaboration. Shark Tank-inspired approaches have been described previously in health professions education [19, 20]. Building on this foundation, EquityRx Tank advances the Shark Tank model in a distinctly different way – shifting the focus from content reinforcement to intentional enrichment, and engaging diverse, interprofessional learners at formative stages of their training.

## Methods

### Curricular Context

EquityRx Tank was developed and implemented as part of a six-week summer enrichment program (SEP) designed to increase knowledge of clinical cancer research, cancer epidemiology, health disparities and professional identity formation across the health professions [21]. The SEP programmatic design included hands-on research experience, clinical practicums, mentorship, and professional development within a robust cancer research environment. EquityRx Tank was a standalone 90-minute session within the science-focused SEP curriculum that provided structured practice in persuasive communication and collaboration skills relevant to healthcare. This session was not part of a longitudinal communication curriculum.

The SEP primarily enrolled undergraduate learners, many of whom self-identified as members of groups underrepresented in medicine and biomedical research. Participants represented varied lived and learning contexts, including racial and ethnic identity (e.g., Black/African American, American Indian or Alaska Native, Native Hawaiian/other Pacific Islander, Hispanic/Latino/a), geographic backgrounds (e.g., urban, suburban, and rural communities across the United States), and first-generation college student status. Learners were organized into five cohorts based on year of SEP enrollment (2019–2023), reaching a total of 71 learners. EquityRx Tank was implemented annually using both in-person (*n* = 61/71; 86%) and virtual (*n* = 10/71; 14%) formats.

## Objectives

The primary objective of EquityRx Tank was to engage learners in persuasive communication and collaboration skills through a learner-centered, game-based instructional model. The session was required for all learners enrolled in the SEP each year. To ensure accessibility and adaptability, no prerequisite readings or assignments were required. Objective competency gains were not discretely assessed; evaluation focused on learner-reported experience related to engagement, perceived enrichment, and collaboration. As a single session, EquityRx Tank was designed to provide structured practice rather than to demonstrate objective competency attainment.

## Instructional Frameworks

**Fig. 1:**
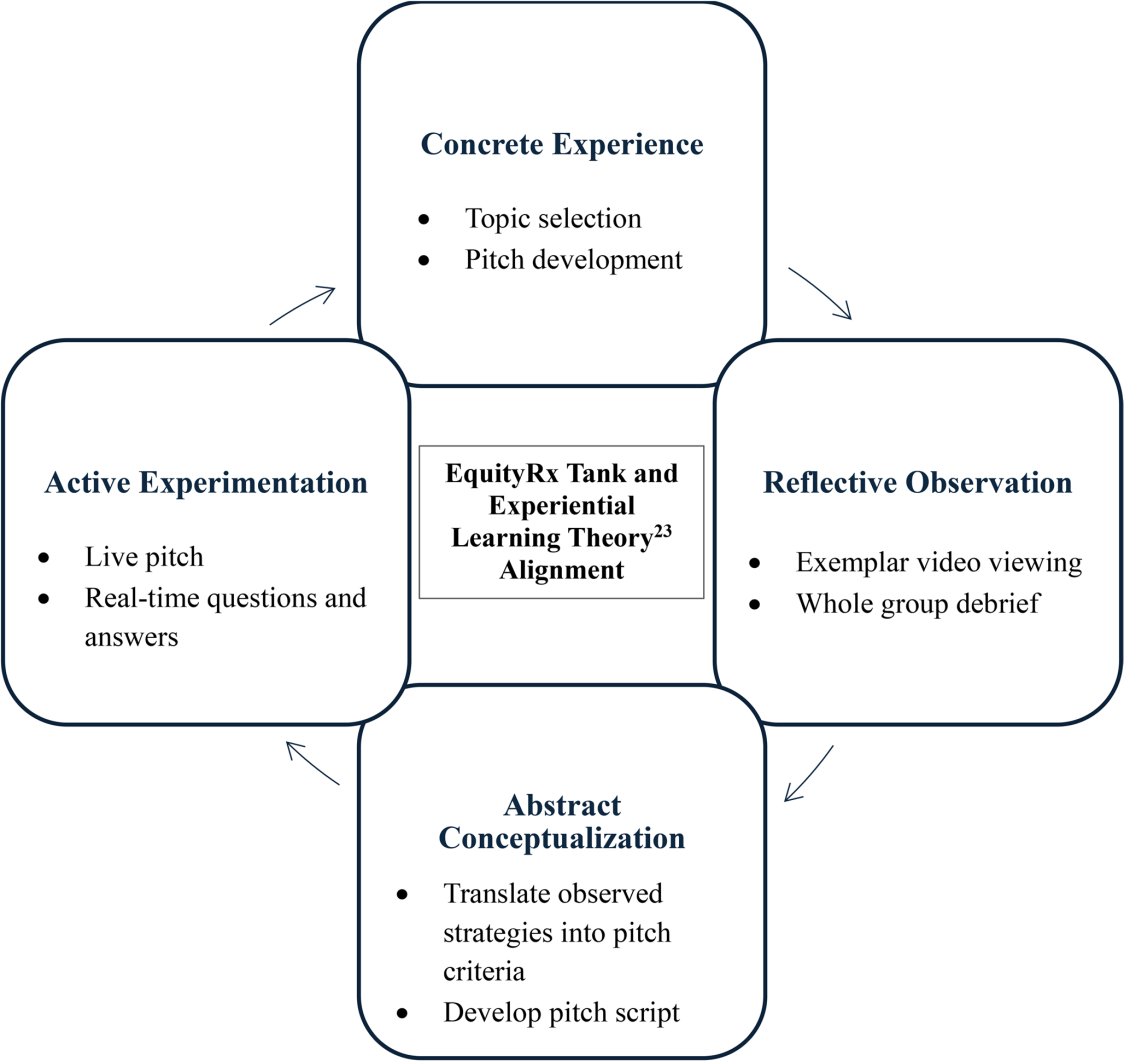
Mapping Experiential Learning Theory to the EquityRx Tank Workshop

Two complementary frameworks informed design and delivery. Understanding by Design (UbD) is a backward design approach guiding identification of enduring goals, development of authentic assessment tasks, and alignment of learning activities [22]. The workshop’s enduring goal was for learners to demonstrate persuasive communication skills while collaboratively addressing a real-world community health challenge. Experiential Learning Theory further informed development, emphasizing the cycle of experience, reflection, conceptualization, and application [23]. We mapped Experiential Learning Theory to the session structure as follows: (1) Concrete experience: small-group topic selection and pitch development; (2) Reflective observation: viewing exemplar videos and whole-group debrief; (3) Abstract conceptualization: linking observed strategies to pitch requirements; and (4) Active experimentation: delivering the pitch and responding to real-time questions and feedback. We anticipated productive discomfort during the pitch presentation, where learners practice the challenge of on-the-spot responses in a supportive, low-stakes setting (Fig. 1).

Working in small groups to design and pitch an innovation required direct experience, guided reflection was facilitated by video exemplars and structured debriefing supported conceptualization and application to future professional contexts. Approximate times are presented for each component. These time allotments were determined pragmatically within the SEP schedule and were not formally pilot-tested or validated; facilitators may adjust timing based on group needs. Every learner was expected to participate in pitch development and to hold a defined role in the final presentation, which served as an observable indicator of participation in the required session.

## Workshop Implementation

Each workshop session lasted 90 min and followed a consistent sequence. As the workshop was required within the SEP, participation expectations were explicit and observable. Each team distributed speaking roles so that all learners contributed verbally during the final pitch.

1. Icebreaker and Reflection (10 min): Learners began with a think-pair-share exercise to brainstorm qualities of memorable, effective public speakers. Whole-group discussion highlighted common strategies for audience engagement, credibility, and persuasion, setting the stage for the workshop focus.
2. Exemplars and Debriefing (15 min): Learners viewed two video exemplars – TED’s Secret to Great Public Speaking and Volkswagen’s Fun Theory Piano Stairs [24, 25]. Facilitators led a structured debrief to link observed principles of persuasion and public communication to strategies for behavior change, highlighting how message structure and audience engagement can motivate action. Videos were selected by the facilitation team for alignment with session objectives and fit within session timing; they were used as instructional exemplars and were not formally validated by subject-matter experts.
3. Group Collaborative Challenge and Pitch Development (35–40 min): Learners worked in small groups (4–5 members) and selected one of three health priorities introduced through newspaper articles highlighting community-identified health needs. By group consensus, one article served as the anchor the group’s presentation. Using structured directions (see Supplement) groups developed a five-minute Shark Tank-style pitch, framing the community health priority as a project proposal seeking funding. Groups incorporated persuasive communication strategies, while being creative and collaborative to address the required elements of the pitch.
4. Pitch Presentation and Judging (25–30 min): Every group pitched their proposal to the larger audience. Facilitators and invited judges evaluated pitches using a rubric (see Supplement) assessing clarity, persuasiveness, feasibility, and collaboration. Judges provided real-time feedback and engaged groups with questions to enhance decision making and critical thinking skills.

Over five years, the workshop was iteratively refined based on learner and facilitator feedback. Adjustments included reducing group size to increase speaking opportunities and incorporating the Volkswagen video in later years to further emphasize innovative thinking and audience engagement [25].

## Evaluation Strategy

Learners completed post-session evaluations via REDCap, including Likert-scale items, a global workshop rating (1 = Poor, 10 = Excellent) and free-text comments. Likert items focused on facilitator behaviors (e.g., clarity of goals, use of materials, engagement) and perceived session value; the judging rubric assessed pitch clarity, persuasiveness, feasibility, and collaboration (tools provided in the Supplement). These instruments were developed for program evaluation and were not formally validated as standardized measures. Program-level demographic data were collected (e.g., race/ethnicity, gender) (Table 1). Written feedback was thematically analyzed, identifying patterns related to satisfaction, engagement, productive discomfort, interprofessional teamwork, and general skill development. Using Braun and Clarke’s reflexive thematic analysis approach, the study team reviewed responses, generated initial codes, and refined themes through discussion to support consistent interpretation [26]. Evaluation activities were approved as part of ongoing programmatic quality improvement and deemed exempt from IRB review.

**Table 1:** Participant characteristics of Summer Enrichment Program (SEP) scholars, 2019-2023 (N=71)

Characteristic	No. (%)
Women (female)	52 (73.2)
First-generation college student	28 (39.4)
Grew up in U.S. rural/low-income and health professional shortage area	44 (62.0)
Underrepresented race/ethnicity in biomedical research/healthcare workforce*	50 (70.4)
**Race**	
Black/African American	22 (31.0)
White	27 (38.0)
Asian	11 (15.5)
Native Hawaiian/other Pacific Islander	1 (1.4)
More than one race	3 (4.2)

*Underrepresented racial and ethnic groups include Black or African American, American Indian or Alaska Native, Native Hawaiian and other Pacific Islander, Hispanic or Latino/a, consistent with National Institutes of Health definitions of underrepresented groups in the biomedical workforce

## Results

### Learner Characteristics

Between 2019 and 2023, 71 learners participated in the EquityRx Tank workshop as part of the SEP. The majority identified as women (73.2%, 52/71). Most learners were near completion of, or had completed, a 4-year undergraduate degree (95.8%, 68/71). 70% (70.4%, 50/71) self-identified as members of racial and ethnic groups underrepresented in the U.S. biomedical research and health care workforce; additional characteristics are shown in Table 1.

### Evaluation Response Rates and Quantitative Findings

Of the 71 learners, 43 (60%) completed the post-session evaluation. Learners rated the workshop highly across all items (Table 2), with a mean global rating of 9.44 on a 10-point scale (1 = Poor, 10 = Excellent). Ratings appeared similar between in-person and virtual cohorts; we did not conduct inferential comparisons due to the unbalanced participant numbers in the in-person and virtual groups.

**Table 2:** Participant Evaluation of the EquityRx Tank Workshop and Facilitators *N* = 43 learners (60%) completed the post-session evaluation

The facilitator(s)…. (1-Poor; 5-Outstanding):	
1. Demonstrated a positive attitude toward teaching and learning	**4.8/5.0**
2. Effectively used teaching materials	**4.8/5.0**
3. Clearly communicated learning goals	**4.7/5.0**
4. Encouraged active engagement	**4.8/5.0**
5. Advanced my understanding of the topic area	**4.7/5.0**
**Overall rating of the workshop facilitator(s)** (1-Poor; 10-Excellent):	
1. Overall, my evaluation of the workshop facilitator(s) is	**9.44/10**

### Qualitative Findings

Thirty-six learners provided written feedback using free text. Five themes emerged from the analysis of learner feedback:

1. Overall Satisfaction. Learners consistently expressed high levels of satisfaction with the workshop. As one learner noted, “This was very interactive and great at facilitating a better understanding of speaking strategies” (2020). Another reflected, “This was such a useful workshop. I’ll continue to work on crafting my [speaking] skills. The TED talk was helpful in providing us with strategies to make information clear and concise. This was genius” (2020).
2. Engagement. Learners emphasized the active, enjoyable nature of the workshop, though increased time was requested. Emphasizing the workshop’s ability to promote participation, one learner shared “…this was a very fun way to get the audience engaged and work on skills simultaneously” (2021). With the level of engagement, the desire for more time was identified, “I wished we had more time to ask questions” (2023) and “I wish we had more time to develop our presentations!” (2023).
3. Productive Discomfort. Several learners acknowledged discomfort with public speaking but framed the learning experience as valuable for their professional growth. One commented, “I struggle with public speaking, and I know this will be an integral part of my career. Having the opportunity to practice with the purpose of refining my speaking skills was great!” (2021).
4. Collaboration. Learners appreciated opportunities for interprofessional teamwork, noting that the workshop fostered meaningful peer interaction. One learner shared, “Very fun activity and opportunity to collaborate with peers and really engage with them” (2023). The observation that smaller groups would be more effective was noted, “I would have liked being in a smaller group” (2021).
5. Skill Development. Learners described gaining concrete skills applicable to future professional contexts and interprofessional environments. One learner explained, “this taught me what considerations need to be made when applying to grants and how to be more efficient with my time” (2023).

### Iterative Workshop Refinements

Learner feedback informed several program adjustments, including reducing group sizes from six members to four to maximize speaking opportunities and adding the Volkswagen video to strengthen connections to behavior change principles [25]. Learners frequently requested more time for preparation and questions and answers, suggesting value in extending the activity to multiple sessions for adequate feedback exchange.

## Discussion

 This study demonstrates the feasibility and value of a Shark Tank-inspired, game-based workshop designed to intentionally cultivate persuasive communication, advocacy, and interprofessional collaboration, competencies often underemphasized in traditional curricula. Implemented across five cohorts over five years, EquityRx Tank engaged learners spanning various backgrounds, fostering collaboration from the outset of training. By embedding these competencies in an interactive, theory-driven format, the workshop offered learners an authentic experience to practice persuasive communication and teamwork in addressing real-world health priorities.

While Shark Tank-inspired models have previously been applied to content mastery and curricular innovation, this study extends the literature by intentionally focusing on competency enrichment rather than content reinforcement. In doing so, EquityRx Tank addresses a recognized gap in professionalism and communication training, responding to calls for structured, reproducible approaches to competency development. Notably, this model provides structured practice in advocacy-related communication (i.e., framing a problem, proposing an action-oriented solution, and tailoring a message to decision makers), which aligns with interprofessional communication expectations across health professions training [11].

Several features distinguish this instructional design. First, the workshop was explicitly theory-driven, grounded in UbD and Experiential Learning Theory, ensuring alignment between instructional goals, activities, and assessment [22, 23]. Making the experiential learning cycle explicit (exemplar reflection, structured pitch development, live practice, and feedback), may help other educators replicate the logic of this design. Second, it was delivered across multiple years and formats, demonstrating adaptability. Third, it intentionally emphasized competency development rather than solely reinforcing content mastery, directly addressing gaps identified in health professions education [7]. Finally, EquityRx Tank extended prior Shark Tank-based innovations by focusing on communication and advocacy skills, engaging undergraduate and interprofessional learners.

While EquityRx Tank demonstrated feasibility and strong learner reception, limitations inform interpretation and opportunities for future development and exploration. The primary limitation relates to the evaluation approach. First, evaluation relied primarily on post-session learner self-report, which may be influenced by response bias, including social desirability and perceived expectations. This limits inferences about skill change because we did not assess baseline public-speaking competencies, prior advocacy/communication training, or learner motivation/engagement, and we did not include objective pre-/post-measures. Future implementations could add brief pre-/post self-efficacy items and a short performance checklist aligned to the rubric. As participation was required within the SEP, we cannot rule out differential engagement related to interest or motivation; we interpret findings as learner reaction and perceived value rather than causal evidence of competency gains.

Additionally, the session utilized exemplar videos on persuasive communication; however, the exemplars were selected for instructional fit and feasibility and were not formally validated by subject-matter experts. Future implementations may strengthen this component through expert review of exemplars or development of context-specific standardized materials. Finally, while iterative refinements were made based on feedback, formal comparisons were not systematically evaluated, and the single-session exposure limits causal claims about sustained impact.

Future work should examine measurable competency gains using feasible objective measures aligned with session objectives and explore longitudinal outcomes related to learners’ ability to advocate, collaborate, and communicate across academic and professional settings. Expansion to additional health professions programs, including medical school clerkships, graduate health programs, and faculty development may broaden its reach and impact. Given its resource-light design and reproducible instructional materials, EquityRx Tank offers a practical model for broader integration.

## Conclusion

EquityRx Tank demonstrates that a Shark Tank-inspired, game-based model can be effectively adapted to foster persuasive communication, advocacy, and interprofessional collaboration competencies in health professions education. Grounded in educational theory and implemented across multiple cohorts of diverse learners, the workshop offers a practical, low-resource, and reproducible framework that complements prior Shark Tank-based innovations by elevating professional competency development rather than content mastery. This model is readily transferable across learner levels and settings and can support the enhancement of essential professionalism competencies that are often underemphasized in traditional curricula. Further, as materials are structured and low-resource, the model can be adapted for interprofessional education, early training experiences, and basic science- or research-focused programs to intentionally pair content learning with structured practice in persuasive communication.

## Supplementary Information

Supplementary Material 1.
