# Supplementary material for: EquityRx Tank: A Shark Tank-Inspired, Game-Based Workshop to Build Persuasive Communication and Collaboration Skills in Health Professions Education
Source: Med Sci Educ. 2026 Feb 27;36(2):795–801. doi: 10.1007/s40670-026-02685-9 (PMC13197561; doi:10.1007/s40670-026-02685-9)
Supplement: Supplementary file 1 — Supplementary Material 1. [file 40670_2026_2685_MOESM1_ESM.docx]

**Structured Directions for Students - EquityRx Tank**

**Congratulations!** Your group has successfully acquired preliminary funding for a project related to the health priority described in your selected newspaper article.

**However,** as in most research projects, funding is limited. Your group must now seek supplemental funding for a revised project component with a proposed budget of approximately $1M.

**Objective:** Develop and deliver a persuasive, five-minute “Shark Tank”-style pitch that clearly communicates your proposed innovation and justifies how supplemental funding will be used.

**Instructions:** Each group will prepare and present a pitch that addresses the following areas, guided by the structured framework below:

1. Carefully read the assigned article to understand the community health priority and your group’s focus.
2. Design a PowerPoint presentation that includes:
   1. A compelling overview of the health priority – begin with a hook to capture the audience’s attention.
   2. A description of the intended community, population, or stakeholder groups most affected.
   3. Specific, measurable objectives for your proposed project.
   4. A detailed plan for how the supplemental funding will be allocated (creativity encouraged!)
   5. A timeline that outlines major milestones
   6. Persuasive elements that appeal to your audience and motivate investment in your idea.
3. Deliver your pitch to the larger group. Each learner must actively participate in the presentation.

**Facilitator Guide - EquityRx Tank**

**Purpose:** To provide a step-by-step guide for facilitating the EquityRx Tank workshop, a Shark Tank-inspired, game-based workshop designed to foster persuasive communication, advocacy, and interprofessional collaboration among health professions learners.

**Preparation (10 minutes before start of workshop)**

- Arrange the room with space for small group collaboration
- Cue video exemplars:
  - TED’s Secret to Great Public Speaking (Chris Anderson) [20]
  - Volkswagen Fun Theory Piano Stairs (YouTube) [21]
- Provide groups with three newspaper articles representing community health priorities.
- Distribute Structured Directions for Learners and Pitch Rubric.

**Workshop (90 minutes)**

1. Icebreaker and Reflection (10 minutes)
   - Prompt: Think of a memorable speaker. What made their communication effective?
   - Use think-pair-share, leading to whole group discussion. Record key qualities.
2. Exemplars and Debrief (15 minutes)
   - Play TED Talk and Volkswagen video
   - Facilitated discussion: highlight hooks, clarity, persuasiveness, audience connection, and advocacy.
   - Connect observations to workshop competencies (persuasive communication, advocacy, interprofessional collaboration).
3. Group Collaborative Challenge (35 minutes)

- Learners form groups of 4-5.
- Each group selects one of three health priorities (from newspaper articles).
- Guided by Structured Directions for Leaners, groups design a 5-minute pitch using PowerPoint.
- Facilitator circulates to answer questions, encourage equitable participation, and reinforce competencies.

1. Presentation and Judging (25 minutes)

- Each group presents to the larger audience.
- Judges use the Pitch Rubric to score and give real-time feedback.
- Allow 2-3 minutes question and answer per group to encourage deeper reasoning.

1. Debrief and Reflection (5 minutes)

- Whole-group reflection: What strategies helped your group be persuasive?

**Facilitation Tips**

- Reinforce creativity in both content and delivery
- Use feedback not only for judging but as a teachable moment.
- Emphasize that skills practiced are transferable to real-world professional advocacy.

**Supplement.** Pitch Rubric - EquityRx Tank

**Project Title: ______________________________________________________________________________**

**Community Health Priority: ______________________________________________________________________________**

**Group Members: ______________________________________________________________________________**

| Criteria | 0  Not Present | 1 Limited | 2  Fair | 3 Excellent | Comments |
| --- | --- | --- | --- | --- | --- |
| All group members demonstrate active engagement and participation during the presentation. |  |  |  |  |  |
| PowerPoint presentation includes all required elements and is visually well-organized. |  |  |  |  |  |
| Proposed project is cohesive, feasible, and addresses the identified need. |  |  |  |  |  |
| Presentation begins with a compelling hook that captures audience attention and motivates investment. |  |  |  |  |  |
| Group members communicate clearly, are articulate, engaging, and persuasive throughout the pitch. |  |  |  |  |  |
